# The Steroids In the Maintenance of remission of Proliferative Lupus nephritis (SIMPL) pilot trial

**DOI:** 10.1186/s40697-014-0030-9

**Published:** 2014-11-28

**Authors:** Lauren Galbraith, Braden Manns, Brenda Hemmelgarn, Michael Walsh

**Affiliations:** Department of Medicine, University of Calgary, Calgary, Canada; Department of Community Health Sciences, University of Calgary, Calgary, Canada; Interdisciplinary Chronic Disease Collaboration, Calgary, Canada; Libin Cardiovascular Institute and Institute for Population Health, University of Calgary, Calgary, AB Canada; Departments of Medicine and Clinical Epidemiology & Biostatistics, McMaster University, Hamilton, Canada; Population Health Research Institute, McMaster University/Hamilton Health Sciences, Hamilton, Canada

**Keywords:** Systemic lupus erythematosus (SLE), Lupus nephritis, Prednisone, Remission, Renal flare

## Abstract

**Background:**

Patients with proliferative lupus nephritis are at risk of frequent relapses. Whether low- dose prednisone prevents relapses is uncertain.

**Objectives:**

We undertook a pilot RCT to determine the feasibility of a larger trial.

**Design:**

Pilot randomized controlled trial.

**Setting:**

Single center Canadian outpatient nephrology clinic.

**Patients:**

Participants with systemic lupus erythematosus (SLE) and a history of class III or IV lupus nephritis that achieved at least partial remission and remained on prednisone were eligible.

**Measurements:**

Feasibility: proportion of eligible patients randomized and adherence to tapering regimen. Clinical: occurrence of renal or major non-renal flare of SLE.

**Methods:**

We conducted a blinded, two-parallel-group randomized controlled trial of prednisone 7.5 mg/day (continuation) compared to a matching placebo (withdrawal).

**Results:**

Of nineteen eligible patients screened, 15 (79%) were recruited and randomized; 8 to prednisone continuation and seven to withdrawal. All participants adhered to the tapering protocol to their assigned withdrawal or low-dose maintenance target. Over 36 months, the primary outcome occurred in four (50%) patients in the continuation group (three renal and one major non-renal flare), compared with one patient (14%) in the withdrawal group (one renal flare). Three participants (38%) in the continuation group had minor flares, while no patients in the withdrawal group did.

**Limitations:**

This pilot RCT was small and not designed to assess the efficacy or safety of maintenance with low-dose prednisone.

**Conclusions:**

The high proportion of eligible patients recruited, and success of protocol adherence suggest a large trial of prednisone maintenance therapy compared to withdrawal is feasible.

**Trial registration:**

Current Controlled Trials ISRCTN31327267.

## Abrégé

**Contexte:**

Les patients atteints de néphropathie lupique proliférative sont sujets à de nombreuses rechutes. Il est incertain que l’administration de faibles doses de prednisone aide à prévenir ses rechutes.

**Objectifs:**

Nous avons entrepris un essai randomisé contrôlé (ERC) (étude pilote) afin de déterminer la faisabilité d’une étude plus vaste.

**Type d’étude:**

Essai randomisé contrôlé (étude pilote).

**Contexte:**

La clinique externe de néphrologie d’un centre canadien.

**Patients:**

Les personnes atteintes de lupus érythémateux disséminé (LED), avec antécédents de néphropathie lupique de stades III et IV, en rémission (minimalement partielle) et toujours en traitement de maintien sous prednisone étaient admissibles à l’étude.

**Mesures:**

Faisabilité : Assignation aléatoire à partir de l’échantillon des patients admissibles et observance de la posologie dégressive.

Clinique : apparition d’une poussée lupique rénale ou d’une poussée lupique grave non rénale.

**Méthode:**

Nous avons mené un essai clinique aléatoire, en parallèle et à double insu, d’un groupe sous 7,5 mg de prednisone (traitement continu) et d’un groupe sous placebo (sevrage).

**Résultats:**

Du bassin des dix-neuf patients triés, 15 d’entre eux (79%) ont été sélectionnés. Le choix du traitement a été attribué de façon aléatoire : 8 patients pour le traitement de maintien sous prednisone et 7 patients pour le sevrage. Tous les participants ont observé le protocole de posologie dégressive qui leur était attribué et dont le but était le sevrage ou le maintien avec de faibles doses de prednisone.

Sur une période de 36 mois, le premier indicateur des résultats est apparu chez quatre patients (50%) du groupe sous prednisone à faible dose (trois poussées rénales et une poussée grave non rénale), comparativement à un patient (14%) du groupe sous sevrage (une poussée rénale). Trois des participants (38%) du groupe sous prednisone à faible dose ont eu des poussées lupiques mineures; aucune poussée lupique mineure n’est apparue dans le groupe sous placebo.

**Limites de l’étude:**

Cet essai randomisé contrôlé pilote a été effectué sur un petit groupe; il n’a pas été conçu pour évaluer l’efficacité ou la sûreté d’un entretien sous prednisone à faible dose.

**Conclusion:**

Le succès combiné du large échantillon de départ de patients admissibles et l’observance du protocole de posologie dégressive laisse suggérer qu’une étude plus vaste comparant le traitement de maintien à la prednisone et le sevrage est faisable.

## What was known before

The duration of prednisone use varies widely after remission is achieved in proliferative lupus nephritis (PLN). Whether prednisone reduces the frequency of flares of PLN is unclear.

## What this adds

A randomized controlled trial comparing long-term low-dose maintenance prednisone to placebo is likely feasible.

## Background

Proliferative lupus nephritis (PLN; class III and IV lupus nephritis) is a potentially organ and life threatening manifestation that affects up to 42% of patients with systemic lupus erythematosus (SLE) [1]. Although over 80% of patients with PLN will initially respond to treatment, many will have relapses that compromise quality of life, worsen kidney function and can be life-threatening.

The prevention of disease relapse is typically accomplished with maintenance of remission therapy, such as immunosuppressants, after remission is induced [2]. Although glucocorticoids are used ubiquitously to induce remission, their use to prevent relapses is heterogeneous and there is very limited data from randomized controlled trials (RCTs) to inform the issue [3,4].

Given the clinical equipoise surrounding the continuation of low-dose glucocorticoids to prevent relapses of SLE and PLN, a RCT is warranted. However, both patients and physicians often have strong opinions regarding both the efficacy and safety of glucocorticoids which can strongly influence a RCTs feasibility [5]. To assess the feasibility of such a trial, we conducted a pilot RCT comparing prednisone 7.5 mg daily to a placebo.

## Methods

We conducted a two-parallel-group randomized controlled trial of prednisone 7.5 mg/day compared to a matching placebo in a single center in Calgary, Canada. The objective of this trial was to assess the feasibility of conducting a larger RCT that would determine the efficacy of long-term low-dose prednisone in patients with a history of PLN. The specific goals of this pilot RCT were to assess our ability to recruit and randomize eligible participants, adherence to a blinded taper and long-term use of a blinded study drug.

Patients were randomly allocated to either the prednisone or placebo group using a random number list generated by an independent statistician. Randomization was blocked and stratified according to the duration of steroid treatment at the time of enrollment (≤12 months or >12 months) and remission status (partial or complete).

Allocation was concealed using sealed, opaque, sequentially numbered envelopes maintained by an independent physician. When a participant was randomized, the independent physician faxed the study number and assigned treatment to the study pharmacy. Patients, investigators, care providers and data analysts remained blinded to study treatment throughout the trial. The University of Calgary research ethics board reviewed and accepted the protocol and all participants provided written informed consent.

### Participants

Eligible patients were at least 18 years of age, had a history of SLE according to ACR criteria [6] and had either class III or class IV lupus nephritis by the ISN/RPS classification system [7,8]. Patients who had class V lupus nephritis in addition to class III or IV were also eligible. Eligible patients must have had an index biopsy within the three years previous to study enrolment, and could have been induced with cyclophosphamide, mycophenolate or another immunosuppressant as seen as appropriate by their physician. Patients were required to be in at least partial remission at the time of randomization (defined as having a) 0.3 to 2.9 g/day proteinuria, b) serum albumin at least 30 g/L and c) stable renal function), be receiving between 5 and 20 mg/day of prednisone and provide informed consent. We excluded patients who were pregnant, required prednisone for treatment of another medical condition other than SLE, or were receiving or expected to receive renal replacement therapy within the next six months.

### Blinding

Blinding of prednisone was accomplished by over-encapsulation of prednisone tablets. Over-encapsulation allowed the groups to receive identical capsules while the dose contained in the capsule for an individual could vary between 1 and 10 mg. Placebo tablets and powder were added to each capsule to ensure the weight of the capsules were identical irrespective of the prednisone dose. Patients presenting mild symptoms of prednisone withdrawal (malaise, nausea, vomiting or hypotension not attributable to another cause) had their prednisone dose increased to the lowest dose achieved without symptom presentation and then reattempted tapering at monthly intervals for a maximum of three attempts. Patients unsuccessful in tapering maintained the lowest dose tolerated for the duration of the trial. Blinding was maintained by instructing the pharmacy to either proceed to the next step of the protocol taper or the previous step of the taper without the study physician knowing what dose was contained at each step. Only the study pharmacy was aware of the actual doses contained at each dose step for each patient. Consequently, participants were unaware as to what dosage of prednisone they were receiving, if any, throughout the trial.

### Interventions

#### Prednisone withdrawal

Patients in the prednisone withdrawal group tapered the dose of prednisone contained in the capsules at a rate of 5 mg/day every two weeks until the dose was 10 mg/day, then by 2.5 mg/day every two weeks until the dose was 5 mg/day and then by 1 mg/day every two weeks until no prednisone and only placebo was contained in the capsules (see Table 1 for complete tapering schedule). A capsule containing placebo only was then continued for the duration of the study.

**Table 1:** **Example of rate of steroid taper for patients allocated to the placebo arm and entering study on 15, 10 and 7.5 mg/day prednisone, respectively**

**Week**	**Prednisone dose (mg/day)**		
1	15	10	7.5
2	15	10	7.5
3	10	7.5	5
4	10	7.5	5
5	7.5	5	4
6	7.5	5	4
7	5	4	3
8	5	4	3
9	4	3	2
10	4	3	2
11	3	2	1
12	3	2	1
13	2	1	0
14	2	1	0
15	1	0	0
16	1	0	0
17	0	0	0

#### Prednisone maintenance

Patients randomized to receive chronic, low-dose maintenance glucocorticoids were tapered from their steroid dose at the time of randomization, if necessary, to a target dose of 7.5 mg/day using the same algorithm as the prednisone withdrawal group. Patients who were already on 5 to 7.5 mg/day of prednisone therapy were maintained on their current dose with no changes made to the dose.

#### Other therapies

Immunosuppressive maintenance therapy was unchanged during the study in the absence of a clinical reason. The use of other therapies including hydroxychloroquine, antihypertensives, non-steroidal anti-inflammatory drugs and HMG CoA reductase inhibitors were left to the discretion of the patients usual care providers. Vitamin D and calcium were recommended for all patients in the trial as osteoporosis prophylaxis. Any patient, regardless of group, with a suspected or confirmed infection received stress dose steroids by the attending physician, which we deemed reasonable given the potential for symptoms of adrenal insufficiency for prolonged periods after glucocorticoid withdrawal [9,10].

#### Assessment and treatment of minor non-renal flares

Physicians were advised to treat minor flares (defined by an increase in Systemic Lupus Erythematosus Activity Index (SLEDAI) score of three points) with a two-week course of 15 mg/day of prednisone. During the treatment of minor flares physicians used open label prednisone. For patients experiencing a minor flare, if there was no clinical response after two weeks of prednisone then the patient had fulfilled the criteria for a major flare and therefore met the pre-defined primary study endpoint.

### Outcomes

Patients were followed for up to 36 months. The feasibility of a larger study was assessed by calculating the proportion of patients screened that were eligible and the proportion of eligible patients that agreed to participate and were randomized. Feasibility was also assessed by evaluating adherence to the blinded tapering protocol by calculating the proportion of participants with at least one protocol deviation.

The primary clinical outcome measure was the composite of renal or major non- renal SLE relapse. A renal relapse was defined as one of any of three events attributed to active SLE (see Appendix 1 for details) 1) a sustained and significant increase in proteinuria; 2) a sustained increase in serum creatinine with new hematuria; or 3) new and sustained glomerular hematuria associated with an increase in proteinuria. A major non-renal flare was defined as either 1) a score greater than nine on the Systemic Lupus Erythematosus Activity Index (SLEDAI) for patients with a baseline score greater than three or, 2) new or worse CNS vasculitis, myositis, platelet count <60,000/mL, anemia with hemoglobin <70 g/L or requirement of prednisone dose >15 mg/day or, 3) hospitalization for SLE or, 4) requirement for new or increased immunosuppressant agent due to disease activity.

Secondary outcome measures included change in health related quality of life measured with the SF-36 energy domain (chosen a priori given that this domain of quality of life might be impacted by withdrawal of prednisone and lupus flares), and the EQ5D index score [11,12]. Exploratory outcome measures included the frequency of adverse events, minor relapses of SLE (i.e. relapses that did not meet the definition of a major relapse) and change in blood pressure over the first12 months.

### Statistical analyses

Feasibility assessment was conducted by calculating the proportion of patients enrolled from the total eligible patients and reported as a frequency (%), protocol violations were calculated and reported in the same way. Baseline patient characteristics are reported as frequency (%) and mean (SD) or median (25^th^ to 75^th^ percentile) as appropriate. For the primary analysis, we compared the time to renal or major non-renal relapse using Cox proportional hazard regression with treatment group as the only independent variable. Analysis time was censored at the time of last follow-up or the occurrence of the primary outcome. The time to first renal or major non-renal relapse was graphically represented with the Kaplan-Meier product limit method. Between group differences in renal and major non-renal relapses, as well as adverse events were assessed using cox regression in which the allocated treatment was the only independent variable. Mixed effects linear regression was used to assess between group differences in SF-36 energy domain, EQ5D index score, and blood pressure to account for repeated measures over time. In these analyses, participants were considered a random effect and treatment group was considered a fixed effect. All analyses were completed with STATA 13 (College Station, Texas, USA).

## Results

**Figure 1:**
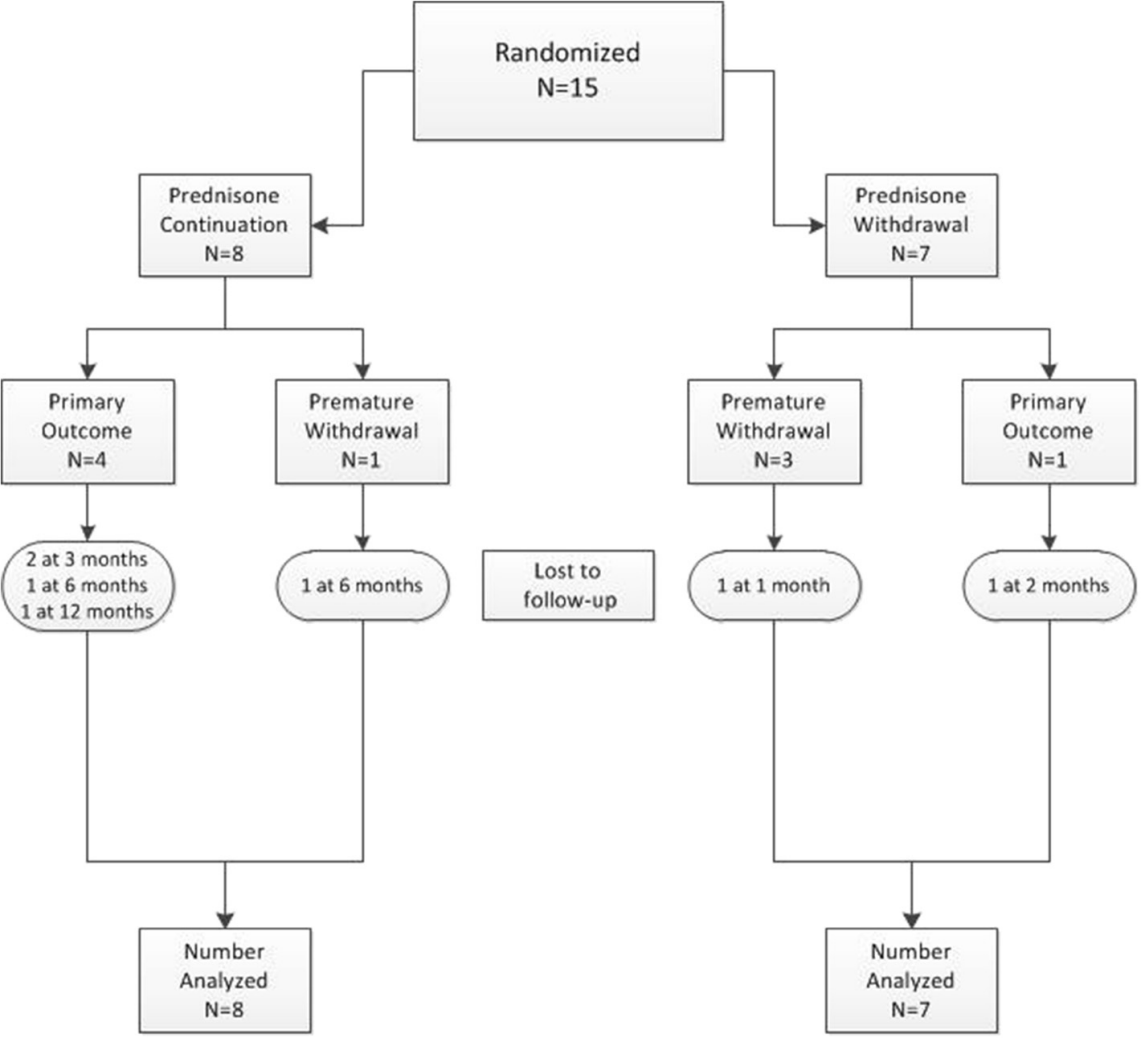
**Participant flow chart.**

We screened fifty-five patients over a 24 month period, of which 19 (34.5%) met eligibility criteria. The largest number of screening failures were either because patients were no longer receiving prednisone (14/36 [39%]) or they were receiving too high a dose (5/36 [14%]). Of the eligible patients, 15 (79%) were enrolled in the study (Figure 1). Eight patients were allocated to prednisone continuation and seven were allocated to prednisone withdrawal. Table 2 summarizes the participant baseline characteristics. The groups differed in mean age and the proportion of patients in complete remission at randomization. The remainder of the characteristics appeared broadly similar.

**Table 2:** **Participant characteristics at randomization**

	**Overall**	**Prednisone continuation**	**Prednisone withdrawal**
	**(n=15)**	**(n=8)**	**(n=7)**
Age (yr), mean (sd)	34.2 (11.2)	39.2 (12.8)	28.4 (5.6)
Female gender, n (%)	13 (86.7)	6 (75)	7 (100)
On steroids >12 months, n (%)	10 (77)	7 (88)	3 (60)
Duration from biopsy to enrollment (days), median (25^th^ to 75^th^ percentile)	342 (463)	495 (497)	331 (548)
Serum creatinine (umol/L), mean (sd)	82.9 (45.4)	85.9 (34.5)	79.4 (58.4)
Proteinuria (g/mmol), mean (sd)	0.07 (0.07)	0.06 (0.07)	0.09 (0.07)
Hemoglobin A1c (%), mean (sd)	5.08 (0.62)	4.8 (0.87)	5.3 (0.2)
Blood Pressure, mmHg (sd)			
Systolic	118.3 (16.3)	125.5 (18.1)	110 (9.4)
Diastolic	73.1 (10.4)	76.3 (10.7)	69.4 (9.5)
Renal Biopsy Class, n (%)^a^			
III	4 (29)	3 (43)	1 (14)
IV	10 (71)	4 (57)	6 (86)
V	8 (57)	3 (43)	5 (71)
Complete remission^b^, n (%)	9 (60)	7 (88)	2 (29)
SLEDAI total score^c^, mean (sd)	1.3 (2.9)	0.25 (0.7)	2.57 (3.9)
SLICC/ACR index^d^, mean (sd)	0.5 (0.9)	0.75 (1.2)	0.29 (0.5)
Prednisone dose (mg/day), mean (sd)	8.1 (1.9)	8.8 (1.6)	7.5 (2.04)
Baseline Cytotoxic Use			
Cyclophosphamide, n (%)	0	0	0
Mycophenolate, n (%)	9 (60)	4 (50)	5 (71)
Azathioprine, n (%)	4 (27)	3 (38)	1 (14)
None, n (%)	2 (13)	1 (12.5)	1 (14)

^a^ISN/RPS International Society of Nephrology/Renal Pathology Society.

^b^Complete remission was defined as having a) less than 0.3 g/day proteinuria, b) normal urine sediment, c) normal serum albumin concentration and d) creatinine value less than 15% above baseline. Partial remission was defined as having a) 0.3 to 2.9 g/day proteinuria, b) serum albumin at least 30 g/L and c) stable renal function.

^c^SLEDAI, Systemic Lupus Erythematosus Activity Index.

^d^SLICC/ACR, Systemic Lupus International Collaborative Clinics/American College of Rheumatology.

Only one patient, in the prednisone withdrawal group, moved away from the study center within the first month, and follow-up data was not available for this patient (Figure 1). Two patients left the study to become pregnant, at 6 months and 18 months. No protocol violations occurred during the tapering regimen. All participants were successfully tapered to their assigned withdrawal or low-dose maintenance target, though one patient in the prednisone continuation group experienced symptoms that were felt to be consistent with prednisone withdrawal. Patients were followed for a median of 11.9 months with a total follow-up of 20.7 person-years.

### Relapses

**Figure 2:**
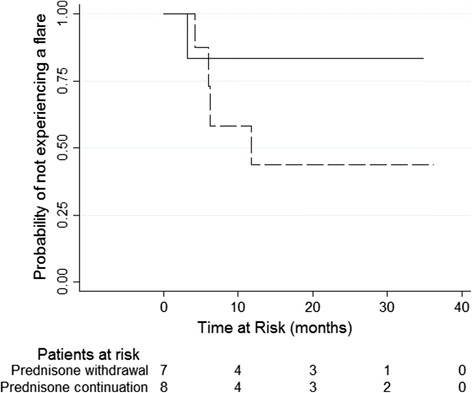
**Survival analysis for renal and non-renal flares.**

Four patients (50%) in the continuation group experienced the primary outcome (three renal flares occurring at 182, 190 and 358 days, and one major non-renal flare at 127 days), compared with one event (14%) (renal flare at 98 days) in the withdrawal group (hazard ratio (HR) 2.68, 95% CI 0.28 to 25.8) (Table 3 and Figure 2). Three participants (38%) had minor flares in the prednisone continuation arm, occurring at 91, 119, and 175 days (including one that progressed to a major non-renal flare, and one that progressed to a renal flare later on), while no patients in the prednisone withdrawal arm experienced minor flares (Table 3).

**Table 3:** **Primary and secondary outcomes across treatment groups**

**Outcome**	**Prednisone continuation**	**Prednisone withdrawal**	**Between groups comparison (HR)**
Renal flare, n (%)	3 (38)	1 (14)	2.68 (0.28, 25.8)
All flares, n (%)	4 (50)	1 (14)	3.35 (0.37, 30.1)
Minor flares, n (%)	3 (38)	0 (0)	a

^a^Hazard ratio could not be computed.

### Other outcomes

There was no significant difference in SF-36 energy scores between the continuation group and the withdrawal group (9.42, 95% CI 4.53 to 19.59). There was also no apparent difference in the change in EQ5D index scores between the continuation group and the withdrawal group (0.67, 95% CI 0.01 to 0.53). Finally, a mixed effects regression analysis for systolic blood pressure, for each patient across all time intervals, found no significant difference comparing the continuation and withdrawal groups (11.54, 95% CI 7.58 to 17.51).

Four participants (50%) in the prednisone continuation group experienced adverse events compared with two (29%) in the prednisone withdrawal group (HR 1.13, 95% CI 0.18 to 6.93). One participant in the prednisone continuation group had two separate episodes of fracture, while two patients developed infections, including one urinary tract infection (Table 4). In the prednisone withdrawal group, one patient had three separate urinary tract infections, and one patient had two separate events of gout and eczema. No patients developed diabetes, and only one patient, in prednisone continuation, developed symptoms consistent with prednisone withdrawal.

**Table 4:** **Participants with adverse events**

	**Prednisone continuation**	**Prednisone withdrawal**
Overall, n (%)	4 (50)	2 (29)
Fracture	_1_a	0
Infection	2	_2_b
New diabetes	0	0
Prednisone withdrawal symptoms	1	0

^a^One patient had both a wrist and foot fracture, independently.

^b^One patient had three separate incidents of UTI.

## Discussion

In this pilot randomized controlled trial of patients with a history of biopsy- proven lupus nephritis in remission, we were able to randomize 79% of eligible patients to withdrawal or continuation of low dose corticosteroids. Given the absence of protocol violations, and adherence to the blinded tapering of prednisone, it is feasible to conduct a large placebo controlled study to determine the efficacy and safety of low-dose prednisone in PLN with this protocol.

To our knowledge, this is the first trial comparing prednisone withdrawal and low-dose prednisone maintenance of remission therapy in patients with a history of PLN. RCTs in rheumatoid arthritis suggests low-dose prednisone modifies disease activity with few side-effects [13]. Early prednisone withdrawal in renal transplantation is associated with more episodes of rejection and in systemic vasculitis early withdrawal is associated with more frequent relapses of disease [14,15]. Finally, small RCTs in SLE suggest moderate doses of prophylactic prednisone attenuate the risk of relapse in patients with a change in the serologic risk markers of relapse. However, enthusiasm for long-term prednisone, even if effective, is tempered by potential side-effects such as infection, gastrointestinal bleeding, and fractures [16]. Together, these data along with substantial practice pattern variation suggest a RCT of long-term low-dose glucocorticoids is warranted.

The main purpose of this pilot trial was to inform the design and conduct of a larger trial that would assess the efficacy and safety of long-term low-dose prednisone in patients with PLN. Such a trial is estimated to require at least 334 patients to detect a 50% reduction in the composite outcome of renal or major non-renal relapse of SLE with 90% power with an average of four years follow-up. Based on our screening and recruitment results, we would require at least 23 similar centers to finish recruitment in 2 years without substantial change to the eligibility criteria. To detect a smaller effect of prednisone much larger sample sizes are required (e.g. hazard ratio 0.75 would require at least 1700 patients). As such, a large trial to answer this question would almost certainly need to be international in scope. However, given how ubiquitously glucocorticoids are used and how important remission maintenance is, such a trial is warranted. The importance of this is underscored by the relative lack of efficacy of many newer adjuvant agents in preventing lupus flares [17,18].

Our pilot RCT has several strengths. We were able to blind both patients and physicians to the withdrawal of prednisone. This improved the objectivity of disease assessment, an inherently subjective process that may be affected by patients and physicians strong underlying beliefs regarding the efficacy of glucocorticoids. We enrolled a broad sample of participants some of which were on very long-term glucocorticoids. This demonstrated that it is possible to withdraw glucocorticoids in these patients in a safe manner and improves the generalizability of the study procedures.

The results of this pilot should be interpreted in light of the study limitations. Our pilot RCT was of insufficient size to assess the efficacy or safety of long-term low-dose prednisone. Even were statistically significant differences in the risk of relapse or adverse events noted in our trial, they would very likely be chance findings [19]. This was a single center experience which limits our confidence that the protocol could be generalized to other centers for a large RCT. However, the process of conducting the pilot informs the design of a larger trial and we believe that such a trial could be further simplified to ensure its feasibility. Also, we chose a dose of 7.5 mg per day of prednisone as our low- dose target. It is unclear if this is the optimal dose in terms of both efficacy and safety and different dosing may also alter the feasibility of the trial. However, there is little data to guide an optimal dose and prednisone 7.5 mg daily represents a commonly low-dose threshold.

## Conclusions

This pilot RCT demonstrated the feasibility of the study protocol. A larger trial comparing prednisone withdrawal and low-dose prednisone as maintenance therapy is possible and is warranted. Any such trial must carefully consider the limited number of patients at each site, as well as individual patients’ complex treatment regimens in that trial’s eligibility criteria and the consideration of the outcome of relapse.

## Appendix 1 Renal flare definition

A renal flare was defined as the occurrence of any one of the three following events:

1. Increased proteinuria, measured by either 24 hour urine collection or by a urine protein to creatinine ratio, by at least a) 1 g/day if the baseline proteinuria was less than 0.2 g/day or, b) 2 g/day if the baseline proteinuria was between 0.2 and 1 g/day (inclusive), or c) more than double the baseline proteinuria if the baseline proteinuria was greater than 1 g/day [20].

2. A sustained (i.e. for two consecutive measures) increase in serum creatinine by at least 30% over baseline that was not due to institution of antihypertensive therapy or angiotensin converting enzyme inhibitor therapy and with new hematuria attributable to active SLE.

3.New sustained hematuria attributable to active SLE, and exclusive of menses, infection or medications, that was associated with an increase in proteinuria by at least 0.8 g/day.

## Abbreviations

SIMPL: Steroids in the maintenance of remission of proliferative lupus nephritis
RCT: Randomized controlled trial
SLE: Systemic lupus erythematosus
PLN: Proliferative lupus nephritis
ACR: American college of rheumatology
ISN/RPS: International society of nephrology/renal pathology society
SLEDAI: Systemic lupus erythematosus disease activity index
CNS vasculitis: Central nervous system vasculitis
SF-36: Short form 36
EQ5D: EuroQol 5D
